# Complete mitochondrial genome sequence of Zhuxiang chicken (*Gallus gallus*. *domesticus*) and its phylogenetic analysis from D-loop region

**DOI:** 10.1080/23802359.2018.1501291

**Published:** 2018-08-13

**Authors:** Li-Li Liu, Ye-Qi Yang, Ya-Ting Liu, Ning Yang, Dan Xie, Zhen Huang

**Affiliations:** School of Life Science, Hunan University of Science and Technology, Xiangtan, China

**Keywords:** Zhuxiang chicken, mitochondrial genome, phylogenetic analysis

## Abstract

Zhuxiang chicken is a valuable chicken breed in the southwest of China. In this study, we firstly obtained the complete mitochondrial genome sequence of Zhuxiang chicken using PCR amplification, sequencing and assembling. The total length of the mitochondrial genome was 16,789 bp, based on composition of 30.24% for A, 23.71% for T, 32.54% for C, 13.51% for G, contained a D-loop region (non-coding control region), 2 ribosomal RNA genes, 13 protein-coding genes and 22 transfer RNA. This research was significant for mtDNA structure and provided phylogenetic analysis of D-loop sequence for carrying out Zhuxiang chicken germplasm resources protection, rational breeding, origin and evolution studies.

Zhuxiang chicken (*Gallus gallus. domesticus*) is mainly spread in Chishui city, which is located in the ‘Hometown of Nanzhu’ in the north of Guizhou Province, China. The chicken ‘Zhuxiang’, having long-term life span in the bamboo forest, has brown-black flat feathers and black-colored skin, meat and bones, and it is a healthy food with high value of nutrition and medicine (Xiao 2012). In this study, the Zhuxiang chicken was obtained from Guizhou Academy of Agricultural Sciences, China. We reported the complete mitochondrial DNA sequence of Zhuxiang chicken (GenBank accession number: KX781318.1). The mitochondrial DNA was extracted using phenol-chloroform protocol (Transgen Biotech, China). There were 22 pairs of primers designed according to the mitochondrial genome sequence of Silky chicken (AB086102.1), and nested polymerase chain reaction was carried out to amplify the complete mitochondrial genome. PCR products of the Gel electrophoresis were purified by Gel AdvancedTM Gel Extraction (Rich Biotech, China) and sequenced by BioSune Biotech (Shanghai, China). DNA sequence was analyzed using the DNAStar7.1 software (Madison, WI). The distribution of mitochondrial DNA sequence and characters of base composition were analyzed using tRNA Scan-SE1.21 and DOGMA software according to previous methods (Liu et al. 2016).

The total length of mitochondrial sequence was 16,789 bp, with the base composition of 30.24% for A, 23.71% for T, 32.54% for C, 13.51% for G in the Zhuxiang chicken. It comprised representative structure, including 1 D-loop region, 2 ribosomal RNA genes, 13 protein-coding genes, and 22 transfer RNA genes. Eight of these mitogenome genes were encoded on the L-strand including tRNA^Cys^, tRNA^Tyr^, tRNA^Ser^, tRNA^Pro^, tRNA^Glu^, tRNA^Gln^, tRNA^Ala^ and tRNA^Asn^, and one protein-coding gene (ND6). There were seven overlaps and 18 spaces in the interval length of 1–10 bp. The initiation codon of protein genes were ATG except for COX1 being GTG. There were four types of termination codon for genes, including TAG for ND2, AGG for COX1, TAA for ND1, COX2, ATPase8, ATPase6, ND3, ND4L, ND5, Cytb and ND6, and an incomplete termination codon ‘T––‘ for COX3 and ND4, ‘T––’ is the 5′ terminal of the adjacent gene, which presumptively formed a complete stop codon by post-transcriptional polyadenylation (Anderson et al. 1981). ATPase6 and ATPase8 were ATP synthase subunit, as important genes regulating the energy synthesis (Mu et al. 2015). Among 13 protein-coding genes, the shortest was 165 bp for ATPase8 between ATPase6 and tRNA^Lys^, while the longest was ND5 (1818 bp) between Cyt b and tRNA^Leu^.

Based on the D-loop sequence region to construct phylogenetic tree, as shown in Figure 1, we could assume that the Zhuxiang chicken has the closest relationship with WPR, Huanglang, and Leghorn chicken, while having a farther distance with *Coturnix chinensis*. This study helps to understand the evolution of *Gallus gallus. domesticus* mitochondrial genome.

**Figure 1.**
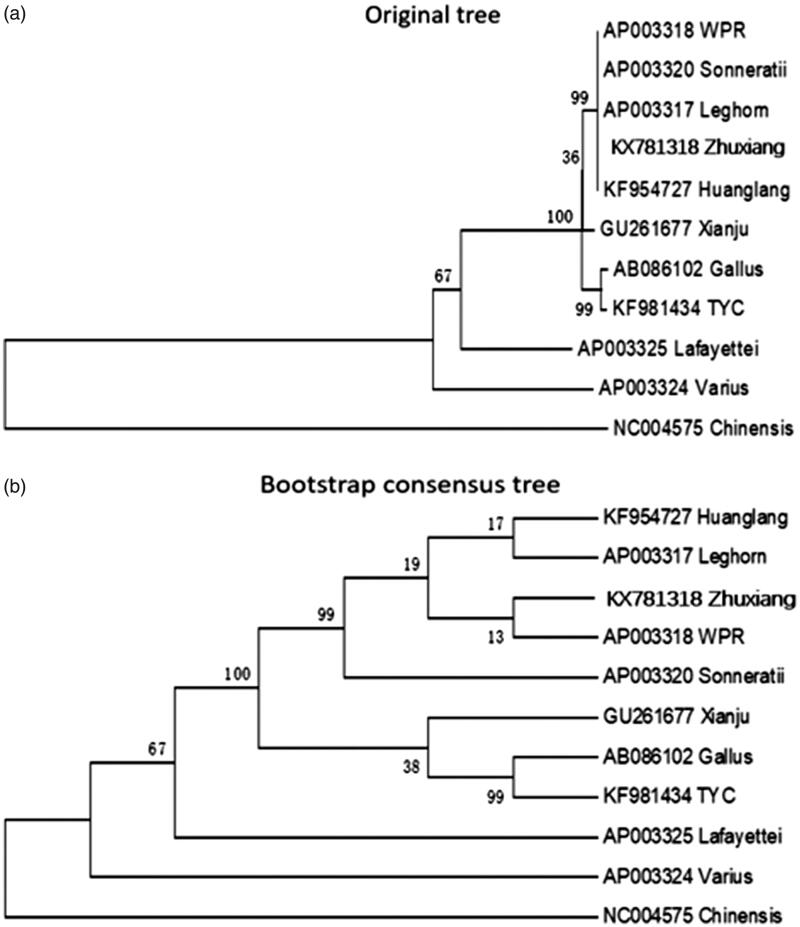
Based on the D-loop sequence to construct phylogenetic tree ((a) Original tree, (b) Bootstrap consensus tree). The mitogenome DNA sequences are downloaded from GenBank and the phylogenetic tree is constructed by a maximum likelihood method by MEGA 5.05. The gene’s accession number for tree construction is listed as follow: Huanglang chicken (KF954727); White leghorn (AP003317); Zhuxiang chicken (KX781318); White plymouth rock (AP003318); *Gallus sonneratii* (AP003320); Xianju chicken (GU261677); *Gallus gallus* (AB086102); Taoyuan chicken (KF981434); *Gallus lafayettei* (AP003325); *Gallus varius* (AP003324); *Coturnix chinensis* (NC004575).
